# Meta-analysis of regional white matter volume in bipolar disorder with replication in an independent sample using coordinates, T-maps, and individual MRI data

**DOI:** 10.1016/j.neubiorev.2017.11.005

**Published:** 2018-01

**Authors:** Stefania Pezzoli, Louise Emsell, Sarah W. Yip, Danai Dima, Panteleimon Giannakopoulos, Mojtaba Zarei, Stefania Tognin, Danilo Arnone, Anthony James, Sven Haller, Sophia Frangou, Guy M. Goodwin, Colm McDonald, Matthew J. Kempton

**Affiliations:** a Department of Neuroscience, Medical School, University of Sheffield, Sheffield, UK; b Department of Psychosis Studies, Institute of Psychiatry Psychology & Neuroscience, King’s College London, UK; c Translational MRI, Department of Imaging & Pathology, KU Leuven, Belgium; d Department of Old Age Psychiatry, University Psychiatry Centre (UPC), KU Leuven, Belgium; e Neuroimaging, Cognition & Genomics Centre (NICOG) & NCBES Galway Neuroscience Centre, College of Medicine, Nursing and Health Sciences, National University of Ireland Galway, Galway, Ireland; f Department of Psychiatry, Yale University School of Medicine, New Haven, CT, USA; g Department of Psychology, City, University of London, UK; h Department of Neuroimaging, Institute of Psychiatry, Psychology and Neurosciences, King’s College London, UK; i Department of Psychiatry, University of Geneva, Switzerland; j National Brain Mapping Centre, Shahid Beheshti University, General and Medical Campus, Tehran, Iran; k Centre for Affective Disorders, Institute of Psychiatry Psychology & Neuroscience, King’s College London, UK; l Department of Psychiatry, University of Oxford, Oxford, UK; m Affidea CDRC – Centre Diagnostique Radiologique de Carouge, Switzerland; n Department of Surgical Sciences, Radiology, Uppsala University, Uppsala, Sweden; o Department of Neuroradiology, University Hospital Freiburg, Germany; p Faculty of Medicine of the University of Geneva, Switzerland; q Icahn School of Medicine at Mount Sinai, New York, USA

**Keywords:** Bipolar disorder, Meta-analysis, VBM, MRI, White matter

## Abstract

Converging evidence suggests that bipolar disorder (BD) is associated with white matter (WM) abnormalities. Meta-analyses of voxel based morphometry (VBM) data is commonly performed using published coordinates, however this method is limited since it ignores non-significant data. Obtaining statistical maps from studies (T-maps) as well as raw MRI datasets increases accuracy and allows for a comprehensive analysis of clinical variables. We obtained coordinate data (7-studies), T-Maps (12-studies, including unpublished data) and raw MRI datasets (5-studies) and analysed the 24 studies using Seed-based d Mapping (SDM). A VBM analysis was conducted to verify the results in an independent sample. The meta-analysis revealed decreased WM volume in the posterior corpus callosum extending to WM in the posterior cingulate cortex. This region was significantly reduced in volume in BD patients in the independent dataset (p = 0.003) but there was no association with clinical variables. We identified a robust WM volume abnormality in BD patients that may represent a trait marker of the disease and used a novel methodology to validate the findings.

## Highlights

• The meta-analysis revealed reductions of white matter volume in the posterior corpus callosum extending to the cingulate cortex.
• The result was replicated in a completely independent dataset.
• There was no association with clinical variables suggesting this may be a trait maker of the illness.

## 1 Introduction

Converging evidence from different MRI modalities suggests that bipolar disorder (BD) is associated with white matter abnormalities. Diffusion tensor imaging meta-analyses in BD have shown fractional anisotropy reduction in clusters located in both anterior and posterior white matter areas (Nortje et al., 2013, Vederine et al., 2011). In addition, meta-analyses of studies using T2 weighted images have confirmed increased rates of deep white matter hyperintensities (WMH) in this disorder (Beyer et al., 2009, Kempton et al., 2008). Meta-analytical data on white matter volume in BD are still limited. Overall, total white matter volume has been found to be preserved (Arnone et al., 2009, Kempton et al., 2008, McDonald et al., 2004b). In terms of regional change, two meta-analyses confirmed a reduction in cross-sectional area of the corpus callosum in BD (Arnone et al., 2008a, Kempton et al., 2008). These findings originate from region of interest (ROI) studies that are restricted to pre-defined areas and so may exclude other regions involved in the illness (Giuliani et al., 2005). Alternatively, voxel-based morphometry (VBM) studies survey the whole brain and examine regions not included in ROI studies (Giuliani et al., 2005, Mechelli et al., 2005). There have recently been a number of meta-analyses of grey matter VBM studies in BD (Bora et al., 2010, Ellison-Wright and Bullmore, 2010, Houenou et al., 2011, Selvaraj et al., 2012, Wise et al., 2016) many of which used published coordinate data (Bora et al., 2010, Ellison-Wright and Bullmore, 2010, Houenou et al., 2011) and one meta-analysis which examined white matter volume using coordinate data from 5 studies (Ganzola and Duchesne, 2017). Meta-analyses of coordinate data are limited because they take into account significant peak findings but ignore sub-threshold results. Two studies (Selvaraj et al., 2012, Wise et al., 2016) have performed a VBM meta-analysis of grey matter using statistical maps (T-maps) in BD. These three dimensional maps comprise statistical data of volume differences in thousands of voxels in the brain. A T-map meta-analysis is more accurate than a coordinate-based meta-analysis, although it requires T-maps to be obtained from the authors of each study (Radua et al., 2012).

We used the software Seed-based d Mapping (SDM) in our meta-analysis of white matter in BD, since it is possible to combine coordinate data, T-maps and even processed raw data (Radua et al., 2012). A growing controversy in scientific research is lack of reproducibility (Editorial, 2016) and this problem has been shown to apply to VBM studies (Boekel et al., 2015). To address this issue we investigated whether the volume reduction identified in our meta-analysis could be replicated in an independent sample. Finally we examined the association between clinical variables and white matter volume in 6 raw MRI datasets comprising 184 BD patients. To our knowledge, our meta-analysis includes the largest number of T-maps of any structural MRI meta-analysis in the bipolar disorder or schizophrenia literature.

## 2 Methods and materials

**Fig. 1:**
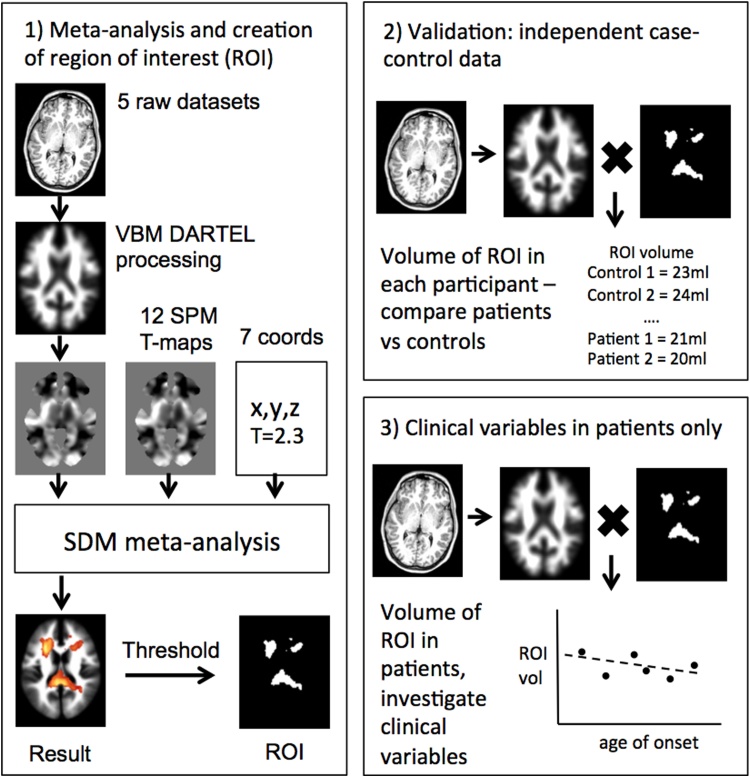
Overview of the three sections of analysis. 1) An ROI in MNI space is created which outlines regions of robust white matter reduction in BD from the meta-analysis. 2) An independent BD dataset is normalised, segmented and modulated using DARTEL and these images are multiplied by the ROI image to give the volume of the ROI in each individual, BD patients are compared to controls. 3) Associations between clinical variables and ROI volume is determined from all available raw patient data.

An overview of the methodology is shown in Fig. 1.

### 2.1 Meta-analysis of white matter VBM studies and creation of ROI

#### 2.1.1 Data source and inclusion criteria of the studies

Articles were obtained from a literature search using the PubMed database. The keywords used were “VBM”, “voxel-based”, “morphometry”, “bipolar”, “mania” and “manic”. An additional manual search within the references section of the articles obtained was also conducted. The studies included were published up to September 2017. Studies were considered if they reported a VBM analysis of white or grey matter volume or density comparing BD patients to healthy controls. A flow chart regarding the selection of the included studies is shown in Fig. s1. Authors of VBM studies were contacted by e-mail asking for their T-map contrast of white matter volume in BD patients compared to controls, and if required, additional information about the associated design matrix to clarify the number of covariates used. To increase the number of studies included in our meta-analysis, authors of studies that only reported grey matter data were contacted to determine whether they had conducted an unpublished white matter analysis. VBM studies were excluded if the study did not report coordinates of white matter changes and if the authors were unable to provide a T-map image or raw MRI data. In studies where separate subgroups of patients were reported, the largest subgroup was used. To ensure that there was no bias to a priori small volume corrections, only studies including whole brain analyses have been considered.

#### 2.1.2 Selection of studies

The initial search retrieved 142 studies of which 81 were eligible. Of the 81, 62 studies reported a grey matter VBM analysis only, and 19 conducted a white matter VBM analysis between BD and controls (Fig. s1). After contacting all of the authors, T-maps of white matter differences between BD patients and controls were obtained from 12 studies including 4 unpublished white matter analyses (Castro-Fornieles et al., 2017, Dukart et al., 2014, Ivleva et al., 2012, Matsubara et al., 2016). In addition a further 5 research groups which published grey matter analyses but did not include VBM white matter analyses, (Emsell et al., 2013, Haller et al., 2011, James et al., 2011, Kempton et al., 2009, Yip et al., 2013) agreed to send us the anonymised individual MRI scans. We subsequently conducted a white matter VBM analysis on these 5 datasets resulting in 5 new T-maps. Therefore, the present meta-analysis included a total of 17 T-maps (Fig. s1). In addition to the T-maps, this meta-analysis included 7 studies for which the peak coordinates were reported (Alonso-Lana et al., 2016, Bond et al., 2014, Farrow et al., 2005, Ishida et al., 2017, McDonald et al., 2005, Stanfield et al., 2009, Watson et al., 2012). The location of each study and the listed authors were compared to determine if there was any possible sample overlap between the studies, and no overlap was identified. Thus the meta-analysis included a total of 24 studies comprising 765 BD patients and 1055 healthy controls.

#### 2.1.3 Creation of T-maps from voxel-based morphometry analysis of 5 raw datasets

Anonymised MRI scans from 5 research groups were processed using SPM8 assessing white matter differences. The VBM pre-processing performed is described below in Section 2 of the methods. Following pre-processing and voxel-wise statistical analysis, a T-map was created from each dataset from the contrast ‘BD patients > controls’ for regional white matter volume. As T-maps include T-scores for every white matter voxel in the brain no threshold was required before including them in the meta-analysis.

#### 2.1.4 Seed-based d mapping analysis

The meta-analysis was performed using the software Seed-based d Mapping (SDM v5.141; available online at http://www.sdmproject.com/) which allows for the combination of statistical maps (T-maps) and peak coordinates (Radua et al., 2012) (Fig. 1). This method has been described in detail by Radua et al. (2012) and the meta-analysis was performed following the instructions available online at www.sdmproject.com/manual/. After receiving T-map images from study authors we verified that the degrees of freedom in each T-map file matched the design matrix reported in the corresponding paper. The T-maps were converted to an unbiased effect size and variance map using the SDM software (Radua et al., 2012). For the studies where only peak coordinates were available, SDM recreated an effect-size signed map (with both positive and negative effect sizes) of the differences in white matter. For each study reporting coordinates, the effect size was exactly calculated within the peaks and was estimated for the other voxels (Radua and Mataix-Cols, 2009, Radua et al., 2012). In order to avoid potential bias from more liberal thresholds applied to particular regions of the brain (e.g. small volume correction), the same threshold was used throughout the entire brain within each study, while different studies were permitted to use different thresholds (Radua, 2015). A z-score map of the pooled effect size was subsequently created by meta-analytically combining each study map, weighted by the inverse variance of each study with between study heterogeneity taken into account (Lansley et al., 2013, Radua et al., 2012, Radua et al., 2011). Thus studies with a larger sample size or less variability contribute more to the pooled effect size (Radua et al., 2012). Statistical significance was determined using a permutation test by means of Monte Carlo randomizations, applying 100 permutations (Radua and Mataix-Cols, 2009, Radua et al., 2011). We used the three thresholds suggested by Radua et al. (2012). The main threshold applied was an uncorrected p value of 0.005 as it this been found to be an optimal balance between sensitivity and specificity, (Radua et al., 2012). Further, to reduce the possibility of false positives, the recommended threshold of z > 1 was applied. Although the z > 1 threshold is usually associated with a non-significant p value under the standard normal distribution, this is not the case under the empirical distribution determined by the permutation tests (Radua et al., 2012). Finally the recommended extent threshold of 10 voxels was applied to exclude smaller clusters. Region names were determined using a white matter atlas (Thiebaut de Schotten et al., 2011). To examine heterogeneity the I^2^ statistic was calculated, which is equal to the percentage of total variation between studies due to heterogeneity. Finally, jackknife sensitivity analyses were conducted in order to assess the reproducibility of the results by repeating the analysis removing one study each time (Radua et al., 2012). In addition, a meta-analysis was performed by including only the 17 studies for which the T-maps were available.

#### 2.1.5 Creation of white matter ROI mask

A region of interest (ROI) mask based on the most significant peaks from the meta-analysis was created by applying a more conservative threshold of z > 3 (Fig. 1). This mask is used in Sections 2.2 and 3.3 below. The ROI was entirely based on the results of the meta-analysis and as such combines more than one anatomically distinct region. The purpose of the ROI is to define a region which most effectively distinguishes patients with bipolar disorder from healthy controls.

### 2.2 Validation of meta-analysis results in an independent VBM study

To independently verify the results of the meta-analysis, a VBM study was conducted using a completely independent sample of BD patients and controls. This additional analysis is reported following the ten rules for VBM studies suggested by Ridgway et al. (2008).

#### 2.2.1 Participants, MRI acquisition and DARTEL analysis

Full details may be found in the supplementary materials. Briefly 26 euthymic patients and 23 closely matched healthy controls were scanned using T1 weighted acquisition and the resulting images were processed using the standard DARTEL (Diffeomorphic Anatomical Registration using Exponentiated Lie algebra) algorithm (Ashburner, 2007) to produce normalised, segmented and modulated images.

#### 2.2.2 Validation: extraction of white matter volumes from ROI defined from meta-analysis

To determine if the most significant results from the VBM meta-analysis could be replicated using this independent dataset, a region of interest (ROI) mask based on the most significant peaks from the meta-analysis was created by applying a threshold of z > 3 to the pooled meta-analysis result. White matter regions defined by the ROI mask were extracted from each smoothed, segmented, normalized, and modulated image from the independent dataset using the MarsBar toolbox for SPM, see Fig. 1. MarsBar returns a single value for each participant which represents their mean voxel value of the ROI. As the segmented data was modulated the mean voxel value was equal to the volume of the ROI in each participant. As the final result was a volume for each participant, rather than voxelwise data, no correction for multiple comparisons was required. The advantage of this method is that it allows a single falsifiable hypothesis to be tested in a new dataset rather than qualitatively comparing clusters of the new dataset to the meta-analysis result. Differences in the ROI volumes between the BD and control groups were assessed with a general linear model (GLM) using total white matter volume as a covariate (SPSS v21).

#### 2.2.3 Examining the association between lateral ventricle volume and the white matter ROI

As the white matter ROI identified in the SDM meta-analysis was adjacent to the lateral ventricles, a region which is known to be enlarged in BD (Kempton et al., 2008), it was important to verify that the ROI decrease was not simply due to this phenomenon. Therefore lateral ventricle volume was determined from the independent dataset using ALVIN (Automatic Lateral Ventricle delIneation, sites.google.com/site/mrilateralventricle) (Kempton et al., 2011). The validation analysis was then repeated using both total white matter volume and lateral ventricle volume as covariates of no interest in the GLM.

### 2.3 Clinical-MRI associations from individual patient data

Investigating the association between clinical and MRI variables in a meta-analysis of case-control studies is challenging because individual patient data are not usually accessible. Meta-regression may be used to analyse study-level data, but this technique is typically low powered. However in the present study we had access to 6 datasets with individual patient data: 5 external datasets (Emsell et al., 2013, Haller et al., 2011, James et al., 2011, Kempton et al., 2009, Yip et al., 2013) and our own dataset. Thus we were able to examine the effect of 6 key clinical variables (age of onset, duration of illness, current lithium use, current antipsychotic use, history of psychosis, BD type) as well as age and gender, on the volume of the region reported in the meta-analysis. Not all clinical variables were recorded at each centre so the number of patients in each analysis was variable as indicated in the results section. Using the ROI mask technique described in the section above, white matter volume was extracted from each BD patient from the 6 VBM processed datasets (184 bipolar patients in total, controls were not included). For each clinical variable, a linear regression analysis was conducted with the clinical measure as the explanatory variable, the ROI white matter volume as the dependent variable, the research centre (a possible confounding categorical variable) and total white matter as a covariate of no interest. For the age of onset and duration of illness, age was additionally included as a covariate of no interest. Standardised coefficients (β) were determined for each regression to indicate the magnitude and direction of the association between the clinical variable and the ROI volume.

## 3 Results

### 3.1 Meta-analysis of white matter VBM studies and creation of ROI

**Fig. 2:**
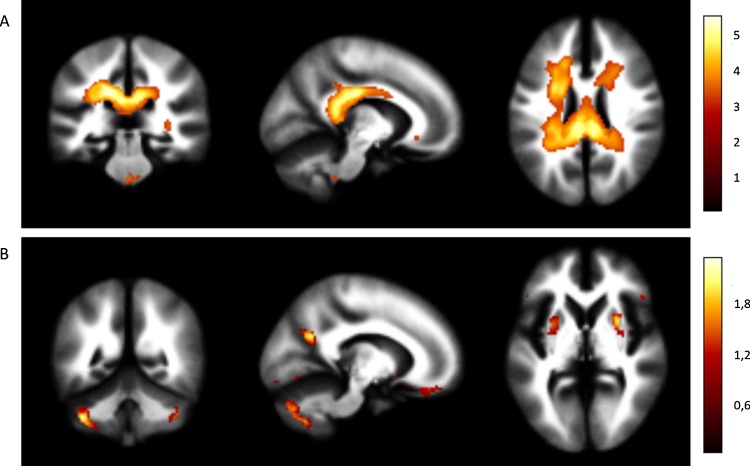
Meta-analysis results showing A) regions of decreased white matter in bipolar patients compared to healthy controls, and B) regions of increased white matter in bipolar patients compared to healthy controls. The colour bars indicates z scores with the standard SDM thresholds applied (p < 0.005 uncorrected, z > 1 and clusters >10 voxels).

The meta-analysis included a total of 24 studies, comprising 765 BD patients and 1055 healthy controls. The demographic and clinical characteristics of the subjects are shown in Table 1. Regions of decreased and increased white matter volume in BD patients compared to controls are shown in Fig. 2 and Table 2. Regions of decreased white matter included a large cluster encompassing the posterior corpus callosum and white matter tracts adjacent to the cingulate gyrus with smaller clusters in the left optic radiation and right frontal superior longitudinal tracts. Regions of increased white matter included small clusters within the cerebellum, and right lenticular nucleus. Heterogeneity was low; I^2^ = 0% for the peak voxel in all clusters except for three clusters where I^2^ < 13% (Table 2). The jackknife sensitivity analyses (Table 2, Fig. s2) that examined the effect of excluding individual studies revealed that the main clusters were highly robust with smaller clusters showing greater sensitivity to excluding individual studies. Results with a Z score > 3 generated the white matter ROI which was used in the sections below. Only regions of white matter reduction in BD were included in the ROI, as white matter volume increases were associated with smaller Z values (Table 2). The results of the meta-analysis performed by including t-maps only are reported in supplementary Table s1.

**Table 1:** Characteristics of the 18 studies included in the meta-analysis.

Study	Patients	Controls	Mean Age of Patients	Mean Age of Controls	Diagnosis of Patients	Mean Age of Onset	Patients Medicated	WM Measure	Covariates	Data
Alonso-Lana et al. (2016)	33	28	44.13 (±SD 6.63)	44.01 (±SD 6.03	Euthymic BDI	NS	NS	Volume	No covariates	Peak Coordinates
Bruno et al. (2004)	39	35	39.1	34.8	28 BDI	25.9	31	Density	No covariates	T-map
			Range 21–63	Range 26–54	11 BDII					
Bond et al. (2014)	57	55	22.8	22.2	Remitted First- Episode Mania BDI	22.8	53	Volume	Age, gender	Peak Coordinates
Castro-Fornieles et al. (2017)	15	70	16.5 (±SD 0.7)	15.3 (±SD 1.5)	Early-onset first episode psychosis	NS	NA	Volume	Age, sex, TIV, scan site were	T-map
Colombo et al. (2012)	26	94	27.12 (±SD 8.47)	30.21 (±SD 8.40)	First Episode Psychosis BDI	26.6	NS	Volume	Gender, total WM	T-map
Dukart et al. (2014)	15	21	NS	47.3 ±9.6	Depressed BD pre ECT	NS	NS	Volume	Age, sex, TIV	T-map
Emsell et al. (2013)	60	60	42 (±SD 10)	42 (±SD 10)	Euthymic BDI	28 (±SD 8)	60	Volume	Age, TIV	Raw data
Farrow et al. (2005)	8	22	F: 17 (±SD 2)	F: 21 (±SD 4)	First Episode Psychosis BDI	NS	NS	Volume	NS	Peak Coordinates
			M: 18 (±SD 2)	M: 20 (±SD 4)						
Haller et al. (2011)	19	47	68.53 (±SD 5.89)	69.77 (±SD 6.55)	Euthymic 10 BDI	39.37 (±SD 15.26)	17	Volume	Age, TIV	Raw data
					9 BDII					
Matsubara et al. (2016)	10	27	46.9 (±SD 12.3)	48.3 (±SD 13.0)	7 BDI	32.2 (±SD 11.5)	10	Volume	Age, sex, premorbid IQ scores	T-map
					3 BDII					
Ivleva et al. (2012)	17	10	38.24 (±SD 7.28)	43.9 (±SD 9.86)	BDI with psychosis	27.7 (±SD 6.3)	16	Volume	Age	T-map
James et al. (2011)	15	20	15.0 (±SD 2.0)	15.3 (±SD 1.0)	BDI with psychosis	14.0 (±SD 2.0)	14	Volume	Age, TIV	Raw data
Kempton et al. (2009)	30	52	39.4 (±SD 9.8)	35.2 (±SD 13.0)	Euthymic BDI	23.5 (±SD 6.7)	27	Volume	Age, TIV	Raw data
Ishida et al. (2017)	29	33	42.7 (SD ±13.3)	37.6 (SD ±9.8)	15 BDI	NA	27	Volume	Age, sex, intracranial volume	Peak Coordinates
					14 BDII					
Matsuo et al. (2012)	35	40	40.8 (±SD 9.2)	41.6 (±SD 9.1)	BDI	19.6 (±SD 10.3)	13	Volume	Age, gend., scan., years ed., hand., total WM	T-map
McDonald et al. (2005)	37	52	40.7 (±SD 11.6)	39.3 (±SD 14.8)	BDI with psychosis	22.9 (±SD 5.5)	32	Volume	Age, gender, global tissue vol	Peak Coordinates
Nugent et al. (2006)	20	65	41 (±SD 8.3)	38 (±SD 11.8)	4 BDI	18 (±SD 8.8)	20	Volume	Age, gender and scanner	T-map
					16 BDII					
Redlich et al. (2014)	58	58	37.5 (±SD 11.0)	37.7 (±SD 9.7)	BD during a depressive episode	23.2 (±SD 9.4)	54	Volume	Age, sex, site	T-map
Rossi et al. (2013)	14	40	43 (±SD 8)	40 (±SD 11)	Euthymic 13 BDI	26	14	Volume	Ed., TIV, abuse alcohol/substance	T-map
					2 BDII					
Sani et al. (2016)	78	78	44.56 (±SD 13.26)	44.38 (±SD 13.31)	49 BDI	NS	78	Volume	Age	T-map
					29 BDII					
Singh et al. (2012)	26	24	15.7 (±SD 1.6)	14.9 (±SD 1.4)	BDI	NS	22 lifetime exposure	Volume	Age, TIV, IQ	T-map
Stanfield et al. (2009)	66	66	36.4 (±SD 11.1)	39.0 (±SD 10.9)	Familial BDI	21.0	NS	Density	Total brain volume	Peak Coordinates
Watson et al. (2012)	24	24	36.0 (±SD 10.0)	35.6 (±SD 9.7)	First Episode Psychosis BD	35.3	NS	Volume	Age	Peak Coordinates
Yip et al. (2013)	34	34	20.94 (±SD 3.16)	21.29 (±SD 2.37)	Bipolar II/NOS disorder	NS	0	Volume	Age, TIV	Raw data

BDI and BDII = Bipolar Disorder I and II; HC = Healthy Controls; FEP = First Episode Psychosis; TIV = Total Intracranial Volume; SD = Standard Deviation; NS = Not Stated; PBD = Pediatric Bipolar Disorder; V = Volume; Ed. = Education; Scan. = Scanner; Gend. = Gender; Hand. = Handedness.

**Table 2:** Meta-analysis results listing regions of decreased and increased white matter in bipolar patients compared to healthy controls. To keep the table to a manageable size we applied an additional criteria of z > 1.5 *Heterogeneity was assessed at each peak voxel using the I^2^ statistic this was 0% for every peak voxel except for L inferior cerebellar peduncle (I^2^ = 12%), R inferior longitudinal fasciculus (I^2^ = 3%) and R corpus callosum tract (I^2^ = 11%), Jackknife shows the number of sensitivity analysis (out of 24) where a result remained significant – the higher the value the more robust the result.

Structure or Tract	Cluster Size	MNI coordinates			SDM Z score (peak voxel)	Uncorrected p value	Hedges g (peak voxel)	Jack-knife (peak voxel)
**Regions of decreased white matter in BD**								
Corpus callosum (left, posterior)	8843	−14	−26	30	5.60	<0.000005	0.23	24
Left optic radiations	250	−30	−70	6	4.21	0.000059	0.21	24
Right frontal superior longitudinal	117	28	−16	56	4.15	0.000077	0.20	24
Left inferior cerebellar peduncle	113	−6	−42	−42	3.75*	0.00047	0.20	23
Left anterior corona radiata	87	−12	30	−10	3.59	0.00092	0.17	22
Right inferior network, inferior longitudinal fasciculus	73	34	−32	2	3.82*	0.00034	0.19	23
Right inferior network, inferior longitudinal fasciculus	62	38	−68	12	3.71	0.00055	0.18	18
Right Corpus callosum tract	10	18	36	20	3.28*	0.0031	0.17	7

**Regions of increased white matter in BD**								
Left cerebellum, hemispheric lobule VIIB	1120	−40	−54	−46	2.31	0.0000018	0.11	24
Left striatum	200	−18	12	−6	1.60	0.00012	0.08	23
Right inferior temporal gyrus, BA 36	188	36	2	−42	1.72	0.000064	0.08	24
Left gyrus rectus, BA 11	152	−8	44	−20	1.86	0.000028	0.09	24
Right striatum	136	26	8	2	1.89	0.000023	0.09	24
Left median network, cingulum	131	−14	−62	24	2.15	0.0000049	0.11	24
Left lingual gyrus, BA 18	126	−20	−78	−12	1.59	0.00013	0.08	24
Right cuneus cortex	130	16	−68	30	1.35	0.00046	0.07	24
Right superior frontal gyrus, medial, BA 8	94	6	36	44	1.40	0.00036	0.07	23
Left inferior frontal gyrus, orbital part, BA 47	75	−36	50	−12	1.48	0.00024	0.07	24
Right superior frontal gyrus, orbital part, BA 11	63	12	46	−20	1.59	0.00013	0.08	24
Left inferior temporal gyrus, BA 37	52	−46	−58	−6	1.91	0.000022	0.09	24
Right supplementary motor area, BA 8	45	6	24	54	1.66	0.000093	0.08	23
Right middle frontal gyrus, BA 46	38	34	46	16	1.53	0.00018	0.07	23

### 3.2 Validation of meta-analysis results in an independent VBM study

Patients with BD and controls did not significantly differ in age, gender, race/ethnicity, weight, height, handedness, premorbid IQ, years of education, lifetime drug and alcohol use or total intracranial volume (Table s2). Clinical characteristics of the BD patients are shown in Table s3. Patients with BD showed a highly significant decrease of the white matter ROI volume (p = 0.0031), validating the findings of the meta-analysis. As the white matter ROI mask generated a single volume for each participant no correction for multiple comparisons was required. When lateral ventricle volume was controlled for, the results remained significant (p = 0.011). For completeness the voxelwise results of the VBM analysis of the independent dataset is shown in Tables s4 and s5 and Fig. s4.

### 3.3 Clinical-MRI associations from individual patient data

Analyses of key clinical variables in the six datasets did not show a significant association between the white matter ROI volume and age of onset (n = 148, β = 0.02, p = 0.68), duration of illness (n = 148, β = −0.02, p = 0.56), lithium use (n = 184, β = −0.02, p = 0.52), antipsychotic use (n = 182, β = 0.00, p = 0.91), history of psychosis (n = 136, β = −0.06, p = 0.34) or bipolar disorder type I (n = 184, β = 0.09, p = 0.081).

In terms of other demographics there was no effect of age (n = 184, β = −0.09, p = 0.066), but a significant effect of gender (n = 184 β = 0.116, p = 0.0002), with males having a smaller ROI as a fraction of total white matter volume. To determine if the gender effect was specific to patients we also examined the association in healthy controls in the six datasets. There was also a significant association (n = 236 β = 0.061, p = 0.017) in the same direction in controls, and no gender x diagnosis interaction (n = 420, p = 0.091). Thus this association appears to be a general effect of gender rather than patient specific.

The meta-analysis result, white matter ROI, T-maps and results from the independent VBM study are available to download from http://www.bipolardatabase.org.

## 4 Discussion

The present study provides evidence supporting regionally specific white matter volumetric abnormalities in BD. The meta-analysis identified a large region of decreased white matter volume that encompassed the corpus callosum and white matter adjacent to the cingulate gyrus. A recent coordinate only based meta-analysis of 5 WM VBM studies (Ganzola and Duchesne, 2017) found 3 small clusters of reduced volume with one cluster in the posterior cingulate. Previous ROI meta-analyses have also reported a reduction of the cross-sectional area of the corpus callosum in BD (Arnone et al., 2008a, Kempton et al., 2008). Consistent with these observations, a recent multicentre study showed significantly decreased cross-sectional area of the posterior corpus callosum in BD compared to controls (Sarrazin et al., 2015). The present voxel-based meta-analysis adds additional detail by revealing white matter volume reductions emanating from this region to white matter adjacent to the posterior cingulate gyrus.

Replication in an independent sample was a particular strength in the present study. Attempting to replicate VBM results are complex because thousands of voxels are involved and previous studies have used the term ‘replication’ to indicate a qualitative similarity of cluster locations (Nenadic et al., 2015). In contrast in the meta-analysis we generated a single falsifiable hypothesis that a combined region of white matter was reduced in bipolar disorder and found strong support for this in the independent sample. We have made the ROI publically available for other research groups to determine if they can replicate this finding. The ROI is not a traditional anatomically defined region, but is defined by the most robust reductions in white matter volume.

Interestingly, the clinical variables in the present study showed no association with the white matter volume ROI in individual patient data, suggesting that the observed white matter decrease is a trait marker of the disease. It is therefore possible that the region is associated with predisposition to BD rather than a manifestation of the illness. An important emerging research focus is the detection of neural abnormalities in those at risk of developing psychiatric disorders. The ability to identify these individuals using neuroimaging would allow subpopulations to be targeted for preventive treatment such as cognitive behavioural therapy (Phillips et al., 2008). White matter alterations, specifically of regional volume and fractional anisotropy, have been found in individuals at genetic risk of BD, such as healthy co-twins and unaffected first-degree relatives suggesting that white matter abnormalities might represent an endophenotype of bipolar disorder (Borgwardt and Fusar-Poli, 2012, Chaddock et al., 2009, Kieseppa et al., 2003, McDonald et al., 2004a). Longitudinal follow-up of those at risk of BD or first episode patients are required to determine if the volume reduction precedes the first symptoms of the illness. From an etiological viewpoint, the absence of associations between the observed MRI alterations and duration of illness or age of onset suggest a neurodevelopmental origin rather than a consequence of the progression of the disease. The demographic variables indicated that males had a comparatively smaller ROI compared to females which was true in both patients and controls and this is likely to be linked to males having a smaller corpus callosum compared to females when correcting for brain size (Ardekani et al., 2013).

The results of our meta-analysis, are consistent with diffusion tensor imaging (DTI) studies that reported fractional anisotropy (FA) reductions in the genu, body and splenium of corpus callosum in BD (Barnea-Goraly et al., 2009, Lagopoulos et al., 2013, Saxena et al., 2012, Wang et al., 2008). In addition, a voxel-based meta-analysis of DTI studies showed decreases of fractional anisotropy in the white matter adjacent the left cingulate gyrus encompassing the middle and posterior cingulum (Nortje et al., 2013). A second DTI meta-analysis found fractional anisotropy reductions in the white matter near the right anterior cingulate cortex and subgenual cingulate cortex (Vederine et al., 2011). Furthermore decreased white matter volumes may be related to the increased prevalence of deep white matter hyperintensities (WMH) which have been observed in BD (Kempton et al., 2008).

The white matter ROI lies adjacent to the posterior cingulate cortex which is a core region of the default mode network (DMN) (Greicius et al., 2003, Raichle et al., 2001). The DMN has been found to be altered in mania (Ongur et al., 2010, Pomarol-Clotet et al., 2012) and decreased connectivity within the posterior DMN has been found in BD patients with psychosis (Khadka et al., 2013). Furthermore Rey et al. (2014) found that BD patients, performing an emotional interference control task, showed a deactivation of the posterior cingulate in hypomania (Rey et al., 2014). Thus our results based on structural MRI data may be linked to findings from resting state and task based fMRI studies in BD.

It is unclear if this abnormality is specific to BD, as reductions in corpus callosum area have also been shown in schizophrenia (Arnone et al., 2008b). Meta-analyses of grey matter VBM studies have shown a remarkably consistent pattern of volume reductions across diagnostic boundaries (Goodkind et al., 2015) and it would be interesting to determine if the pattern of white matter volume reduction observed in BD is also present in other disorders. Future research into white matter abnormalities in BD could integrate information from different neuroimaging techniques such as structural MRI, DTI tractography and T2 weighted imaging of hyperintensities to study not only volumetric changes in white matter, but also the anatomical connectivity between regions and their relationship to focal hyperintensities.

Several limitations should be taken into account when interpreting the present results. We combined studies with different populations of patients, and variations in MRI acquisition and analysis techniques leading to increased heterogeneity, however this also ensures the results are representative of the current literature. VBM has more frequently been used in identifying regional grey matter changes, however VBM meta-analyses of white matter have been conducted in schizophrenia, (Di et al., 2009) autism (Radua et al., 2011) and Alzheimer’s disease (Li et al., 2012). White matter volume provides limited information regarding neuropathology and DTI is likely to be a better measure of white matter integrity. However, white matter volume has advantages as it is less sensitive to motion than DTI and is not affected by artifacts caused by crossing fibers (Le Bihan et al., 2006). Furthermore to interpret DTI changes correctly it is important to know if there are volumetric changes in white matter. We cannot exclude that white matter hyperintensities contributed to the volume loss reported in the posterior corpus callosum and adjacent areas near the lateral ventricles. However, this is an unlikely scenario since it has been previously shown that although deep white matter hyperintensities are increased in BD, this is not the case for periventricular hyperintensities (Kempton et al., 2008). We were not able to obtain T-maps for 7 studies and used published coordinates instead. Including coordinates increased the number of studies but may have increased heterogeneity. Although the combination of T-maps and coordinates using SDM has been well validated (Radua et al., 2012), we performed the meta-analysis excluding those 7 studies, obtaining overlapping results.

To our knowledge, our meta-analysis includes the largest number of t-maps of VBM studies included within the bipolar disorder, and schizophrenia literature. We also provide both white and grey T-maps from our own study for future meta-analyses. Our work complements other meta-analytical frameworks and consortiums such as ENIGMA (van Erp et al., 2015) which have begun large-scale neuroimaging analyses of psychiatric populations, principally using FreeSurfer to examine anatomically defined ROIs. We have made the region from the meta-analysis available online (http://www.bipolardatabase.org) which will allow other investigators to determine if they can also replicate the findings reported here.

In conclusion, the present study demonstrates the presence a regional white matter volume reduction in BD, adding further evidence of abnormalities of white matter within this patient group. Further investigation of this region from MRS, DTI and postmortem data may clarify the neuropathological origin of these changes and longitudinal studies of those at risk of developing BD may clarify when these changes first occur.

## Financial/conflict of interest disclosures

Prof Goodwin holds a grant from Wellcome Trust, holds shares in P1vital and has served as consultant, advisor or CME speaker for AstraZeneca, Merck, Cephalon/Teva, Eli Lilly, Lundbeck, Medscape, Otsuka, P1Vital, Pfizer, Servier, Sunovion, Takeda.

All other authors report no conflict of interest.

## Responsibility of data analysis

Stefania Pezzoli and Matthew Kempton had full access to all the data in the study and take responsibility for the integrity of the data and the accuracy of the data analysis.
