# Supplementary material for: Meta-analysis of regional white matter volume in bipolar disorder with replication in an independent sample using coordinates, T-maps, and individual MRI data
Source: Neurosci Biobehav Rev. 2018 Jan;84:162–70. doi: 10.1016/j.neubiorev.2017.11.005 (PMC5771263; doi:10.1016/j.neubiorev.2017.11.005)
Supplement: Supplementary file 1 [file mmc1.docx]

**Supplementary Materials**

**Supplementary Methods**

**Independent VBM study**

**Subjects**

The VBM analysis included 26 euthymic patients with BD (23 with bipolar I and 3 with bipolar II, 9 males and 17 females) and 23 healthy control subjects (7 males and 16 females). The patients were primarily recruited from a UK patient support group, healthy controls were recruited via advertisements in local media. The study was approved by the local ethics committee and written informed consent was obtained from all participants. All subjects were assessed using the Structural Clinical Interview for DSM-IV Axis I Disorders (SCID-CV). Patients were included if they fulfilled criteria for DSM-IV for BD and did not have any comorbidity for other DSM-IV Axis-I disorders. Healthy controls subjects were selected in order to match BD patients for age, sex, race/ethnicity, weight, height, handedness, premorbid IQ, years of education, lifetime drug and alcohol use. They were included if they had no DSM-IV Axis I disorders and no family history of psychiatric conditions. The mean age was 42.1 (± SD 14.8) for BD patients and 41.2 (± SD 14.0) for healthy controls. Demographic and clinical measures are given in table s1 and s2.

**MRI acquisition**

Participants were scanned using a 1.5 Tesla Siemens Magnetom Vision MRI scanner to obtain T1 weighted MPRAGE (Multi-Planar Rapidly Acquired Gradient Echo) scans. In order to confer good resolution and good contrast between grey and white matter in particular, the following parameters were selected: TR = 9.7 ms, TE = 4 ms, TI = 300 ms, Nex = 1, 256 x 192 matrix, flip angle = 8°, 128 slices, voxel size = 1.0 x 1.0 x 2.0mm. There was no significant difference in scan date between patients and controls (p=0.28).

**VBM DARTEL pre-processing**

We examined group-related differences in regional brain volume using voxel-based morphometry, as implemented in SPM8 software (<http://www.fil.ion.ucl.ac.uk/spm/>) running under MATLAB R2012b, version 8.0 (The MathWorks, Icn, Natick, Massachussetts). First the T1-weighted images were pre-processed using the DARTEL (Diffeomorphic Anatomical Registration using Exponentiated Lie algebra) algorithm (Ashburner, 2007) following the steps described by Ashburner (Ashburner, 2010). Firstly, each T1-weighted image was checked for scanner artefacts and gross anatomical abnormalities and then manually reoriented to the Anterior Commissure-Posterior Commissure line blind to diagnosis. The images were then segmented into grey matter, white matter and cerebrospinal fluid in native space. The DARTEL SPM8 toolbox was used to implement the high-dimensional DARTEL normalization through which the DARTEL template was created from the images of all the subjects of the study. During the template creation, flow fields were computed which contain information about the transformation from every native image to the DARTEL template (Peelle et al., 2012). This procedure increases the accuracy of the alignment between subjects by using millions of parameters to characterise the spatial transformations of each brain (Ashburner, 2010). In order to allow for inter-study comparisons, the segmented images were spatially normalized to MNI space including the flow fields in the process. The images were ‘modulated’ to conserve the information on absolute volume. Smoothing was applied to the images using a FWHM 8mm isotropic Gaussian kernel resulting in smoothed, segmented, normalized, and modulated images.

**VBM Statistical analysis**

A central aim of the study was to examine the volume of the white matter ROI created by the meta-analysis in an independent sample, however for completeness in the supplementary materials we present the whole VBM brain analysis of the independent dataset. Total intracranial volume was determined for each subject by summing grey matter, white matter and CSF segmentations. The regional differences in voxel-based parameters between BD and controls were assessed using a General Linear Model (GLM) with total intracranial volume and age as covariates of no interest. An absolute threshold masking of 0.05 was adopted in order to exclude voxels outside the brain. A height threshold of p < 0.05 FWE (family wise error) corrected was initially adopted to detect significant regional differences. In addition a more liberal height threshold of p < 0.001, uncorrected for multiple comparisons, was also applied with a cluster threshold of 10 voxels. Following this height threshold, a non-stationary cluster extend correction was implemented at the *cluster* threshold of p < 0.05 family-wise error (FWE) corrected for multiple comparisons in order to account for the non-isotropic (non-uniform) smoothness across the data (Hayasaka et al., 2004; Worsley et al., 1999). This correction was performed using the VBM8 toolbox (available online at <http://dbm.neuro.uni-jena.de/vbm/download>). Finally we implemented the same method excluding patients who were taking lithium as studies have demonstrated that lithium may increase total grey matter volume (Hallahan et al., 2011; Kempton et al., 2008; Monkul et al., 2007; Moore et al., 2009; Sassi et al., 2002). Montreal Neurological Institute (MNI) coordinates are reported in the results tables (supplementary table 3 and table 4), however these coordinates were converted to Talairach coordinates to determine the names of corresponding brain regions. MNI coordinates were converted to Talairach using GingerALE, version 2.1.1 (available online at http://www.brainmap.org/ale/) and brain region names were determined using Talairach Client, version 2.4.3 (available online at http://www.talairach.org/client.html).

**Supplementary Results**

**Independent VBM whole brain study results**

No significant differences in white or gray matter volume were found at the height threshold of p < 0.05 FWE corrected. The analysis was then repeated with a height threshold of p < 0.001 uncorrected. Regions of significant white matter volume decreases at a height threshold of p<0.001 uncorrected are shown in supplementary table 3. No regions of significant increased white matter in bipolar patients compared to controls were found. Two clusters of voxels survived the additional non-stationary *cluster* extent threshold of p < 0.05 FWE corrected for multiple comparisons in the white matter results. These clusters encompassed white matter adjacent to the cingulate gyrus and in the corpus callosum (supplementary figure 3). Grey matter volume differences between the two groups are also shown in supplementary table 3. Finally, we found regions of decreased and increased grey matter in bipolar patients that were not taking lithium compared to healthy controls (supplementary table 4). The T-maps of each contrast are freely available to download from [www.bipolardatabase.org](http://www.bipolardatabase.org). The white matter results have been used in the main paper to validate the region of interest found in our meta-analysis.

**Supplementary Tables**

| **Structure or Tract** | **Cluster Size** | **MNI coordinates** | | | **SDM**  **Z score**  **(peak voxel)** | **Uncorrected p value** |
| --- | --- | --- | --- | --- | --- | --- |
| Regions of decreased white matter in BD | | | | | | |
| Corpus callosum (left, posterior) | 7766 | -4 | -38 | 20 | 6.36 | <0.0001 |
| Left optic radiations | 304 | -30 | -70 | 6 | 5.09 | <0.0003 |
| Left inferior network, inferior fronto-occipital fasciculus | 150 | -40 | -40 | 4 | 4.31 | 0.00038 |
| Left precentral gyrus, BA 6 | 148 | -36 | -12 | 54 | 4.63 | 0.00012 |
| Corpus callosum | 118 | -12 | 30 | -10 | 4.21 | 0.00053 |
| Left inferior cerebellar peduncle | 90 | -6 | -42 | -42 | 4.23 | 0.00051 |
| Right inferior network, inferior longitudinal fasciculus | 77 | 38 | -68 | 12 | 4.12 | 0.00074 |
| Right inferior network, inferior longitudinal fasciculus | 54 | 34 | -34 | 2 | 4.23 | 0.00050 |
| Corpus callosum (genu) | 38 | 2 | 28 | 0 | 3.78 | 0.0021 |
| Right superior longitudinal fasciculus III | 25 | 46 | -20 | 32 | 3.94 | 0.0013 |
| Left inferior network, inferior longitudinal fasciculus | 12 | -34 | -52 | -6 | 3.85 | 0.0018 |
| Right superior longitudinal fasciculus III | 11 | 38 | -4 | 20 | 3.80 | 0.0021 |
| Regions of increased white matter in BD | | | | | | |
| Left cerebellum, hemispheric lobule VIIB | 1074 | -40 | -54 | -46 | 2.80 | 0.0000016 |
| Left striatum | 224 | -24 | 8 | 0 | 2.08 | 0.000066 |
| Right inferior temporal gyrus, BA 36 | 166 | 36 | 2 | -42 | 2.08 | 0.000065 |
| Right striatum | 126 | 26 | 8 | 2 | 2.29 | 0.000023 |
| Left gyrus rectus, BA 11 | 131 | -8 | 44 | -20 | 2.25 | 0.000028 |
| Left median network, cingulum | 119 | -14 | -62 | 24 | 2.6 | 0.0000052 |
| Left lingual gyrus, BA 18 | 112 | -20 | -78 | -12 | 1.92 | 0.00014 |
| Right superior frontal gyrus, medial, BA 8 | 112 | 6 | 36 | 44 | 1.70 | 0.00038 |
| Left striatum | 86 | -32 | 52 | -8 | 1.87 | 0.00017 |
| Right inferior temporal gyrus, BA 20 | 71 | 60 | -32 | -16 | 1.76 | 0.00029 |
| Right cuneus cortex | 66 | 16 | -68 | 30 | 1.64 | 0.00050 |
| Left inferior temporal gyrus, BA 37 | 49 | -46 | -58 | -6 | 2.30 | 0.000021 |
| Right supplementary motor area, BA 8 | 41 | 6 | 24 | 54 | 1.87 | 0.00018 |
| Right middle frontal gyrus, BA 46 | 41 | 36 | 48 | 14 | 1.89 | 0.00016 |
| Corpus callosum | 38 | 26 | -64 | 30 | 1.59 | 0.00062 |
| Right gyrus rectus, BA 11 | 35 | 10 | 48 | -20 | 1.67 | 0.00044 |
| Right inferior frontal gyrus, triangular part, BA 45 | 31 | 50 | 26 | 4 | 1.71 | 0.00036 |
| Left lingual gyrus, BA 18 | 31 | -18 | -94 | -14 | 1.34 | 0.0017 |
| Left inferior frontal gyrus, triangular part, BA 45 | 26 | -48 | 36 | -2 | 1.66 | 0.00044 |
| Left middle frontal gyrus, BA 8 | 25 | -24 | 18 | 48 | 1.65 | 0.00046 |
| Right inferior frontal gyrus, triangular part, BA 48 | 25 | 46 | 24 | 28 | 1.42 | 0.0012 |
| Left temporal pole, superior temporal gyrus, BA 21 | 22 | -48 | 4 | -18 | 1.36 | 0.0015 |
| Right postcentral gyrus, BA 3 | 19 | 34 | -36 | 52 | 1.56 | 0.00067 |
| Right lingual gyrus, BA 18 | 18 | 16 | -90 | -14 | 1.52 | 0.00081 |
| Left inferior frontal gyrus, opercular part, BA 44 | 18 | -44 | 16 | 32 | 1.48 | 0.00097 |
| Right lingual gyrus, BA 18 | 19 | 14 | -68 | -8 | 1.31 | 0.0018 |
| Left middle frontal gyrus, orbital part, BA 11 | 16 | -18 | 54 | -16 | 1.56 | 0.00069 |
| Right inferior temporal gyrus, BA 37 | 16 | 48 | -54 | -8 | 1.39 | 0.0013 |
| Left inferior frontal gyrus, triangular part, BA 45 | 15 | -52 | 26 | 8 | 1.52 | 0.00082 |
| Left precentral gyrus, BA 6 | 14 | -50 | 0 | 38 | 1.57 | 0.00067 |
| Left postcentral gyrus, BA 43 | 14 | -60 | -14 | 32 | 1.41 | 0.0013 |
| Left fusiform gyrus, BA 20 | 13 | -34 | -14 | -26 | 1.42 | 0.0012 |
| Right middle occipital gyrus, BA 19 | 12 | 30 | -82 | 22 | 1.52 | 0.00080 |
| Right middle occipital gyrus, BA 7 | 12 | 32 | -76 | 38 | 1.48 | 0.00096 |

**Supplementary Table s1 Meta-analysis of T-maps only.** Regions of decreased and increased white matter in bipolar patients compared to healthy controls resulted from the meta-analysis including 17 t-maps only (Co-ordinate data is not included).

| **Characteristic** | **Control Subjects (n=23)** | **Bipolar Patients (n=26)** | **Statistic** | **p value** |
| --- | --- | --- | --- | --- |
| Age  Mean (± SD) | 41.2 (± 14.0) | 42.1 (± 14.8) | t_(47)_ = 0.23 | p = 0.82 |
| Sex  M:F | 7:16 | 9:17 | Fisher’s Exact Test | p = 1.00 |
| Race  White | 21 | 26 | Fisher’s Exact Test | p = 0.21 |
| Height in metres  Mean (± SD) | 1.70 (± 0.08) | 1.70 (± 0.09) | t_(47)_ = 0.13 | p = 0.90 |
| Weight in kilograms Mean (± SD) | 78.6 (± 17.9) | 78.5 (± 21.4) | t_(47)_ = 0.016 | p = 0.99 |
| Right handed | 20 | 23 | Fisher’s Exact Test | p = 1.00 |
| NART IQ  Mean (± SD) | 116.3 (± 6.5) | 117.7 (± 5.1) | t_(47)_ = 0.80 | p = 0.43 |
| Years of education Mean (± SD) | 16.8 (± 2.9) | 15.4 (± 3.6) | t_(47)_ = 1.54 | p = 0.13 |
| Individuals with lifetime drug use | 1 | 6 | Fisher’s Exact Test | p = 0.10 |
| Individuals with lifetime alcohol abuse | 1 | 3 | Fisher’s Exact Test | p = 0.61 |
| Total Intracranial Volume (ml)  Mean (± SD) | 1531.31  (± 131.59) | 1570.46  (± 156.04) | t_(47)_ = 0.94 | p = 0.35 |

SD = Standard Deviation.

NART = National Adult Reading Test.

**Supplementary Table s2** Demographic characteristics for bipolar patients group and controls group. There were no significant differences among the groups.

| Bipolar I/Bipolar II | 23/3 |
| --- | --- |
| Age of onset | 26.0 (± SD 11.3) |
| Length of illness | 16.2 (± SD 12.8) |
| Hamilton Rating Scale for Depression | 3.2 (± SD 2.4) |
| Young Mania Rating Scale | 2.0 (± SD 2.7) |
| Number of Hospitalizations | 3.0 (± SD 2.3) |
| Patients previously treated with ECT | 6 |
| Mood stabilizer | 16 (62%) |
| - Lithium | 9 (35%) |
| - Sodium Valproate | 5 (19%) |
| - Carbamazepine | 5 (19%) |
| Antipsychotic medication | 4 (15%) |
| Antidepressant | 11 (42%) |
| - SSRI | 5 (19%) |
| Hypnotic | 5 (19%) |
| Drug free | 5 (19%) |

**Supplementary Table s3** Clinical features of bipolar patients included into the study and medication taken by bipolar patients included into the study. 35% of patients were taking only one type of medication.

| **Structure** | **Cluster Size** | **MNI coordinates** | | | **T_(45)_ score** | **Uncorrected p value**  **(peak voxel)** |
| --- | --- | --- | --- | --- | --- | --- |
| Regions of decreased white matter volume | | | | | | |
| WM adj to Right Posterior Cingulate* | 3277 | 9 | -39 | 24 | 4.48 | < 0.001 |
| WM adj to Right Cingulate Gyrus* | 3498 | 5 | 12 | 25 | 4.25 | < 0.001 |
| WM adj to Left Cingulate Gyrus | 143 | -3 | -15 | 36 | 4.13 | < 0.001 |
| WM adj to Left Superior  Frontal Gyrus | 23 | -15 | 60 | 25 | 4.07 | < 0.001 |
| WM adj to Right Claustrum | 72 | 30 | 29 | -2 | 3.98 | < 0.001 |
| WM adj to Left Middle  Occipital Gyrus | 35 | -36 | -72 | 13 | 3.81 | < 0.001 |
| WM adj to Left Anterior Cingulate | 231 | -2 | 30 | 1 | 3.70 | < 0.001 |
| WM adj to Left Lentiform Nucleus | 30 | -27 | -9 | -12 | 3.49 | 0.001 |
| WM adj toRight Caudate | 22 | 6 | 5 | -6 | 3.47 | 0.001 |
| Regions of decreased grey matter volume | | | | | | |
| Right Claustrum | 256 | 30 | 30 | 7 | 4.04 | < 0.001 |
| Left Posterior Cingulate | 204 | -6 | -34 | 25 | 3.98 | < 0.001 |
| Left Inferior Frontal Gyrus | 24 | -44 | 36 | -5 | 3.82 | < 0.001 |
| Regions of increased grey matter volume | | | | | | |
| Declive  Right Cerebellum | 54 | 17 | -72 | -11 | 4.16 | < 0.001 |
| Culmen  Left Cerebellum | 71 | -2 | -69 | -2 | 3.91 | < 0.001 |
| Left Rectal Gyrus  Frontal Lobe | 21 | -6 | 45 | -35 | 3.71 | < 0.001 |
| Declive  Right Cerebellum | 21 | 15 | -82 | -20 | 3.51 | 0.001 |
| Cerebellar Tonsil  Left Cerebellum | 18 | -14 | -63 | -42 | 3.46 | 0.001 |

**Supplementary Table s4** Regions of decreased and increased white and grey matter volume in bipolar patients compared to healthy controls in the independent dataset (p < 0.001 uncorrected height threshold). *= survives *cluster* level p<0.05 FWE corrected for multiple comparisons.

| **Structure** | **Cluster Size** | **MNI coordinates** | | | **T_(36)_ score** | **Uncorrected p value**  **(peak voxel)** |
| --- | --- | --- | --- | --- | --- | --- |
| Regions of decreased grey matter volume | | | | | | |
| Right Insula | 13 | 28 | 30 | 9 | 3.52 | 0.001 |
| Right Anterior Cingulate | 52 | 9 | 45 | -17 | 3.52 | 0.001 |
| Left Anterior Cingulate | 19 | 0 | 26 | 15 | 3.49 | 0.001 |
| Left Posterior Cingulate | 22 | -8 | -36 | 25 | 3.48 | 0.001 |
| Regions of increased grey matter volume | | | | | | |
| Left Postcentral Gyrus | 201 | -68 | -6 | 13 | 4.31 | < 0.001 |
| Left Postcentral Gyrus | 28 | -62 | -22 | 46 | 3.79 | < 0.001 |
| Culmen  Left Cerebellum | 53 | -2 | -69 | -2 | 3.77 | < 0.001 |
| Left Superior  Temporal Gyrus | 49 | -47 | 9 | -20 | 3.64 | < 0.001 |
| Left Superior  Temporal Gyrus | 16 | -56 | 15 | -9 | 3.48 | 0.001 |

**Supplementary table s5** Regions of decreased and increased grey matter volume in bipolar patients that were not taking lithium compared to healthy controls in the independent dataset (p < 0.001 uncorrected height threshold)

**Supplementary Figures**

**
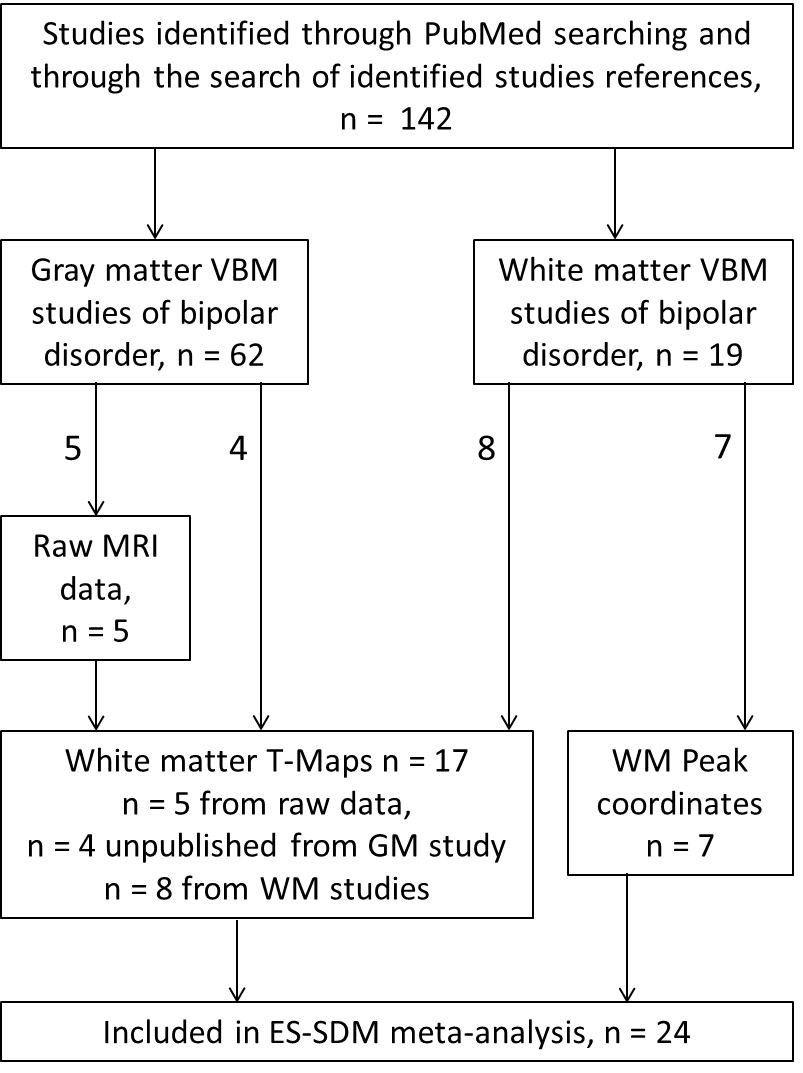
**

**Supplementary Figure s1** Flow chart showing the selection of the included studies.


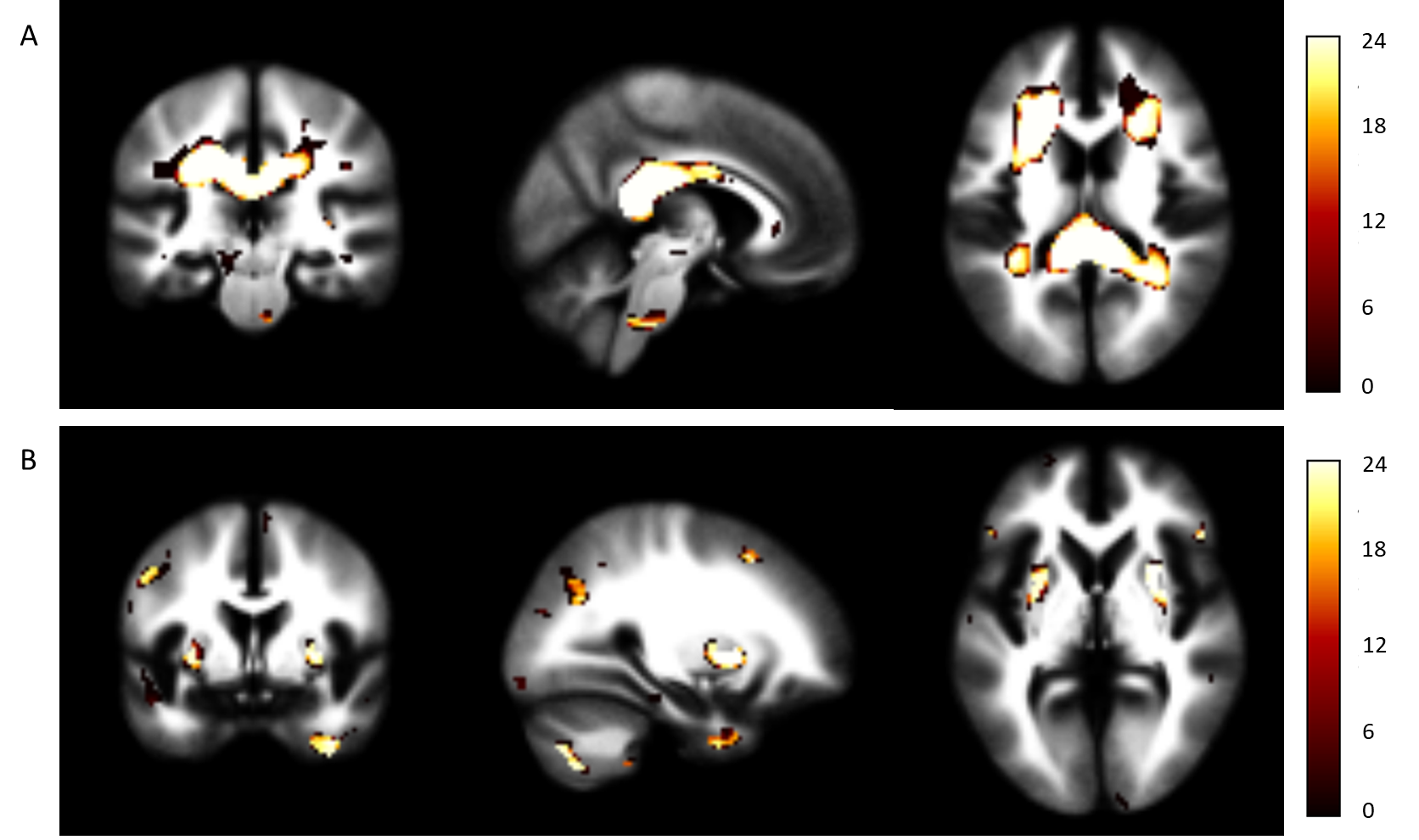


**Supplementary Figure s2** Binarised maps of jackknife sensitivity analyses representing A) regions of decreased white matter in bipolar patients, and B) regions of increased white matter in bipolar patients. The colour bars indicate the number of overlapping jackknife maps. The colour indicates the number of sensitivity analysis (out of 24) where a result remained significant. E.g. a white voxel represents a value of 24, and indicates that the region was significantly reduced in volume in all 24 of the sensitivity analyses each study in turn was excluded.


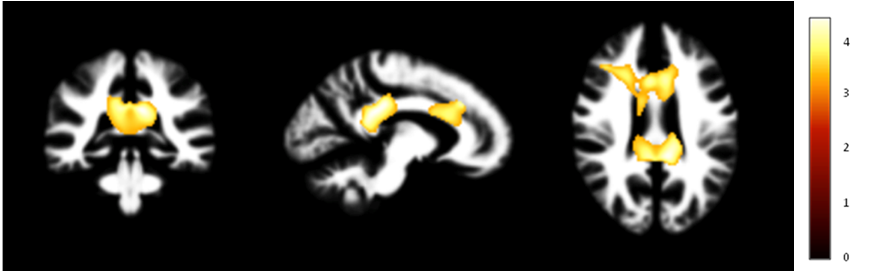


**Supplementary Figure s3** Regions of significantly decreased white matter in bipolar patients compared to healthy controls in the independent dataset, overlaid on the white matter DARTEL template created in the present study with an height threshold of p < 0.001 uncorrected for multiple comparisons and a non-stationary cluster extend corrected threshold of p < 0.05 FWE corrected for multiple comparisons.

**References for Supplementary Methods**

Ashburner, J., 2007. A fast diffeomorphic image registration algorithm. NeuroImage 38, 95-113.

Ashburner, J., 2010. VBM tutorial.

Hallahan, B., Newell, J., Soares, J.C., Brambilla, P., Strakowski, S.M., Fleck, D.E., Kieseppa, T., Altshuler, L.L., Fornito, A., Malhi, G.S., McIntosh, A.M., Yurgelun-Todd, D.A., Labar, K.S., Sharma, V., MacQueen, G.M., Murray, R.M., McDonald, C., 2011. Structural magnetic resonance imaging in bipolar disorder: an international collaborative mega-analysis of individual adult patient data. Biological psychiatry 69, 326-335.

Hayasaka, S., Phan, K.L., Liberzon, I., Worsley, K.J., Nichols, T.E., 2004. Nonstationary cluster-size inference with random field and permutation methods. NeuroImage 22, 676-687.

Kempton, M.J., Geddes, J.R., Ettinger, U., Williams, S.C., Grasby, P.M., 2008. Meta-analysis, database, and meta-regression of 98 structural imaging studies in bipolar disorder. Archives of general psychiatry 65, 1017-1032.

Monkul, E.S., Matsuo, K., Nicoletti, M.A., Dierschke, N., Hatch, J.P., Dalwani, M., Brambilla, P., Caetano, S., Sassi, R.B., Mallinger, A.G., Soares, J.C., 2007. Prefrontal gray matter increases in healthy individuals after lithium treatment: a voxel-based morphometry study. Neuroscience letters 429, 7-11.

Moore, G.J., Cortese, B.M., Glitz, D.A., Zajac-Benitez, C., Quiroz, J.A., Uhde, T.W., Drevets, W.C., Manji, H.K., 2009. A longitudinal study of the effects of lithium treatment on prefrontal and subgenual prefrontal gray matter volume in treatment-responsive bipolar disorder patients. The Journal of clinical psychiatry 70, 699-705.

Peelle, J.E., Cusack, R., Henson, R.N., 2012. Adjusting for global effects in voxel-based morphometry: gray matter decline in normal aging. NeuroImage 60, 1503-1516.

Sassi, R.B., Nicoletti, M., Brambilla, P., Mallinger, A.G., Frank, E., Kupfer, D.J., Keshavan, M.S., Soares, J.C., 2002. Increased gray matter volume in lithium-treated bipolar disorder patients. Neuroscience letters 329, 243-245.

Worsley, K.J., Andermann, M., Koulis, T., MacDonald, D., Evans, A.C., 1999. Detecting changes in nonisotropic images. Human brain mapping 8, 98-101.
